# Accuracy of multimodal intravascular imaging in evaluating neointimal tissue in a swine femoropopliteal artery stent model

**DOI:** 10.1007/s12928-026-01321-y

**Published:** 2026-07-29

**Authors:** Kazuki Aihara, Sho Torii, Norihito Nakamura, Manabu Shiozaki, Yu Sato, Tsukasa Kato, Yuki Matsumoto, Yuji Ikari, Gaku Nakazawa

**Affiliations:** 1 Department of Cardiology, Tokai University School of Medicine, 143 Shimokasuya, Isehara, 259-1193 Kanagawa Japan; 2 Department of Cardiology, Kindai University Faculty of Medicine, Sakai, Japan

**Keywords:** Peripheral artery disease, Endovascular treatment, Intravascular imaging, Stent failure, Drug-eluting stents

## Abstract

Stent failure, including in-stent restenosis (ISR) and occlusion, remains a major challenge in femoropopliteal arteries. Since the underlying cause of stent failure, such as fibrin-rich thrombus versus organized fibrous plaque, likely dictates the optimal treatment strategy, accurate characterization of neointimal tissue is crucial. However, pathological validation for key intravascular imaging modalities in this setting is lacking. A total of 22 femoral arteries with an in-stent restenosis model in healthy swine after stent implantation were evaluated. One month later, intravascular imaging using intravascular ultrasound (IVUS), optical frequency domain imaging (OFDI), and angioscopy was performed. Diagnostic accuracy for characterizing neointimal tissue ≥ 1000 μm (thick neointima) was assessed using histology as the gold standard. For detecting fibrin/organizing thrombus, IVUS showed high specificity (96%) but poor sensitivity (13%), leading to underestimation. Conversely, OFDI offered relatively high sensitivity (60%) but a modest positive predictive value (PPV, 50%), risking overestimation. A paired use of IVUS and OFDI approach overcame these limitations, yielding significantly improved diagnostic accuracy (sensitivity 50%, specificity 96%, PPV 83%). While angioscopy showed excellent specificity (100%), its utility was limited by very low sensitivity (24%). Paired use of IVUS and OFDI may improve diagnostic confidence for neointimal tissue characterization. In contrast, the utility of angioscopy appears limited due to low sensitivity.

**Graphical abstract:**

**Figure:**
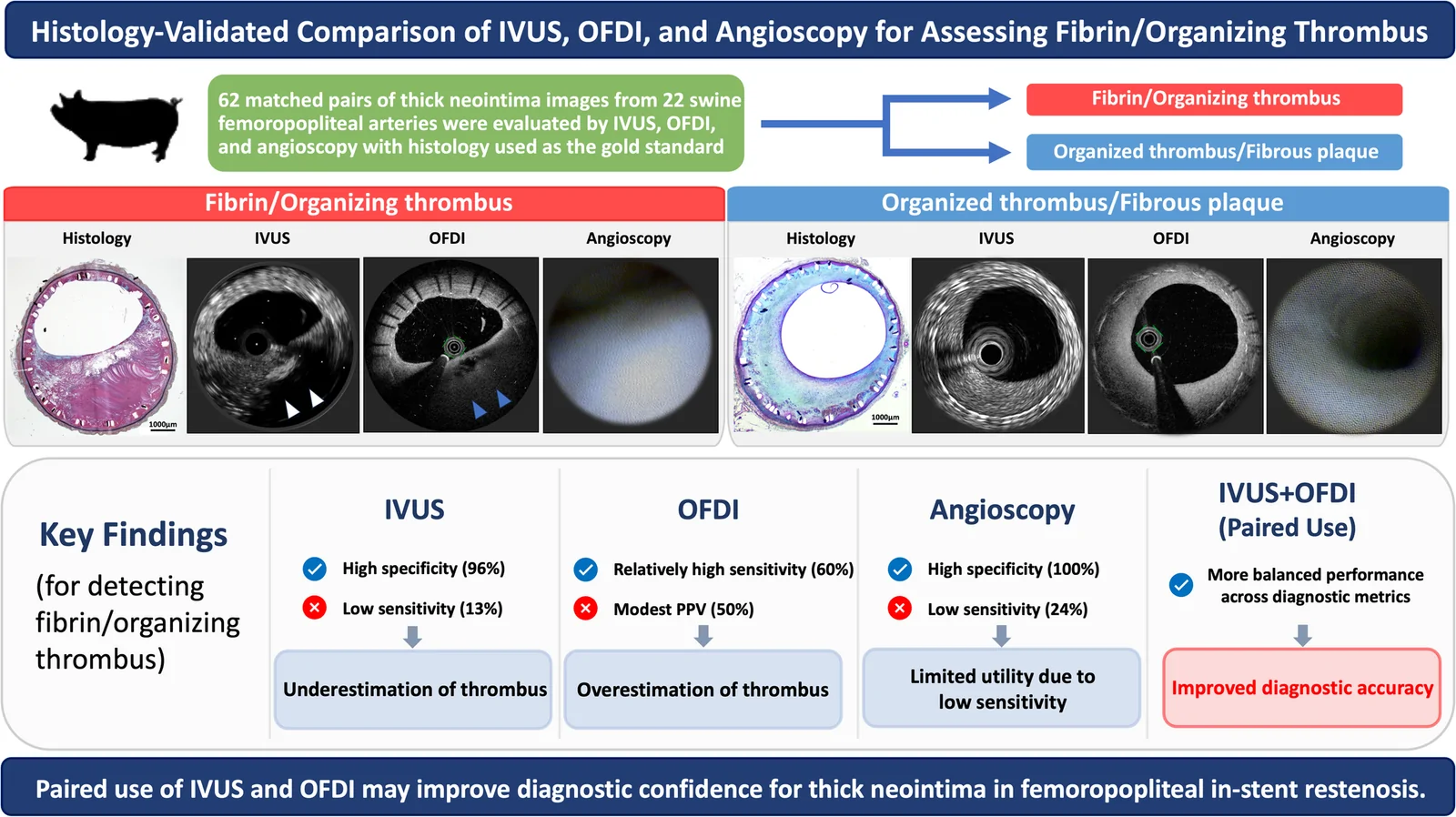

**Supplementary Information:**

The online version contains supplementary material available at https://doi.org/10.1007/s12928-026-01321-y.

## Introduction

The global prevalence of lower extremity artery disease (LEAD) in adults is estimated at 9.7%, and LEAD is a growing global health concern, leading to a significant increase in both endovascular therapy (EVT) and surgical bypass procedures [1–3]. In recent years, EVT has become the primary treatment strategy for femoropopliteal lesions, with clinical outcomes improving due to advancements in drug-eluting stents (DES) and drug-coated balloons (DCB) [2, 4]. The long-term clinical outcomes of femoropopliteal DES remain suboptimal compared to those in coronary artery interventions [5]. Consequently, stent failure, which encompasses both in-stent restenosis (ISR) and occlusion, continues to be a significant clinical challenge.

A critical aspect of addressing stent failure is recognizing that its underlying pathology is not uniform. The composition of neointimal tissue can vary, ranging from organized thrombus and fibrous plaque to fibrin-rich deposits [6]. This difference is clinically significant, as the optimal treatment strategy may depend on the specific tissue characteristics. For instance, lesions rich in smooth muscle cells may respond well to DCB, whereas those composed primarily of fibrin or organizing thrombus might be better suited for treatment with excimer laser atherectomy or mechanical thrombectomy [7–9]. Moreover, identifying a significant thrombotic burden may also guide post-procedural pharmacological strategy, such as the addition of direct oral anticoagulants to mitigate the risk of acute limb ischemia [10]. Therefore, an accurate assessment of neointimal characteristics using intravascular imaging is crucial for diagnostic characterization of stent failure.

Despite the clinical need for accurate tissue characterization, the diagnostic accuracy of key intravascular imaging modalities has not been pathologically validated in femoropopliteal arteries. While a previous study compared findings from computed tomography (CT) with histology, no study to date has performed a histology-controlled comparison of common intravascular modalities like intravascular ultrasound (IVUS), optical frequency domain imaging (OFDI), and angioscopy [6]. Thus, this study aimed to evaluate the accuracy of multimodal intravascular imaging in characterizing neointimal tissue in femoropopliteal lesions by comparing these imaging modalities with histological findings.

## Methods

### Study framework

The present study was conducted as part of a broader preclinical swine femoropopliteal stent project that originally included 18 swine (36 femoropopliteal arteries) [11, 12]. The overall project was designed to investigate vascular responses after implantation of different stent platforms—including bare nitinol stents and drug-eluting stents—in a healthy swine model with poor below-the-knee runoff. Within this experimental framework, lesions were categorized according to neointimal thickness. The current analysis specifically focused on lesions with thick neointima (≥ 1000 μm) to assess the ability of IVUS, OFDI, and angioscopy to characterize neointimal tissue composition in developed in-stent lesions.

### Animal model and stent implantation procedure

All animals received care in compliance with the Animal Welfare Act and Public Health Services policies. The in-stent restenosis model was created in healthy swine as previously described [11]. In brief, swine were administered dual-antiplatelet therapy starting two days before the procedure. Under general anesthesia, a 6-F guide catheter was advanced into the common femoral artery, and coils (VortX-18 Diamond coil, Boston Scientific, Marlborough, MA, USA) were placed in the tibial artery to induce poor below-knee runoff. Subsequently, one of three types of 6.0 × 40 mm stents (bare nitinol stents [Innova; strut thickness about 220 μm; Boston Scientific, Marlborough, MA, USA], fluoropolymer-coated paclitaxel-eluting stents [Eluvia; drug dose 0.167 µg/mm², strut thickness about 220 μm; Boston Scientific, Marlborough, MA, USA], or polymer-free paclitaxel-eluting stents [Zilver PTX; drug dose 3.0 µg/mm², strut thickness about 220 μm; Cook Corporation, Bloomington, IN, USA]) was implanted in each femoral artery. The animals were monitored daily for one month.

### Vessel harvesting and preparation

One month after the procedure, follow-up angiography was performed to evaluate the vascular state. The swine were then euthanized via intravenous injection of pentobarbital and/or potassium. The stented vessels were excised, pressure-perfused with 0.9% saline to clear all blood, and subsequently fixed by perfusion with 10% formalin. Post-fixation radiographs confirmed full stent expansion without fractures.

### Ex vivo intravascular imaging acquisition

After formalin fixation, the stented arteries were imaged ex vivo while immersed in a 0.9% saline bath. An imaging run was performed from the distal to the proximal segments using IVUS (AltaView, Terumo, Tokyo, Japan), OFDI (LUNAWAVE, Terumo, Tokyo, Japan), and angioscopy (VISIBLE; FiberTech, Chiba, Japan). IVUS images were acquired at a pullback rate of 0.5 mm/s, and OFDI images at 20 mm/s.

### Intravascular imaging analysis

All imaging data were analyzed at our institution. Every 1 mm slice of the stented segment was evaluated. Based on previous reports, neointimal tissue characteristics were qualitatively assessed [13,14–17]. For IVUS, hypoechoic areas in the neointima were classified as fibrin/organizing thrombus, while isoechoic areas were classified as organized thrombus/fibrous plaque. For OFDI, signal-poor, highly attenuated lesions with diffuse borders were defined as fibrin/organizing thrombus, whereas lesions with homogeneous backscattering, low attenuation, and visible stent struts were defined as organized thrombus/fibrous plaque. For angioscopy, the color of the neointima covering the stent struts was evaluated. Neointimal color was classified into two categories: red and white neointima [18]. Red neointima was defined as fibrin/organizing thrombus, whereas white neointima was defined as organized thrombus/fibrous plaque. Yellow plaque grading was not included in the present analysis because lipid-rich atheromatous lesions were not observed histologically in this healthy swine model. All images were reviewed by two experienced investigators (N.N. and S.T.) who were blinded to the histological findings. For the paired IVUS+OFDI assessment, the final classification was determined by a consensus interpretation that integrated the complementary information of both modalities, rather than by a simple either-positive or both-positive rule. Quantitative measurements were also obtained from ex vivo IVUS and OFDI images in the thick neointima group. Stent area and lumen area were manually traced on each cross-sectional image. Neointimal area was calculated as stent area minus lumen area, and percent area stenosis was calculated as neointimal area divided by stent area × 100.

### Histological processing and analysis

**Fig. 1:**
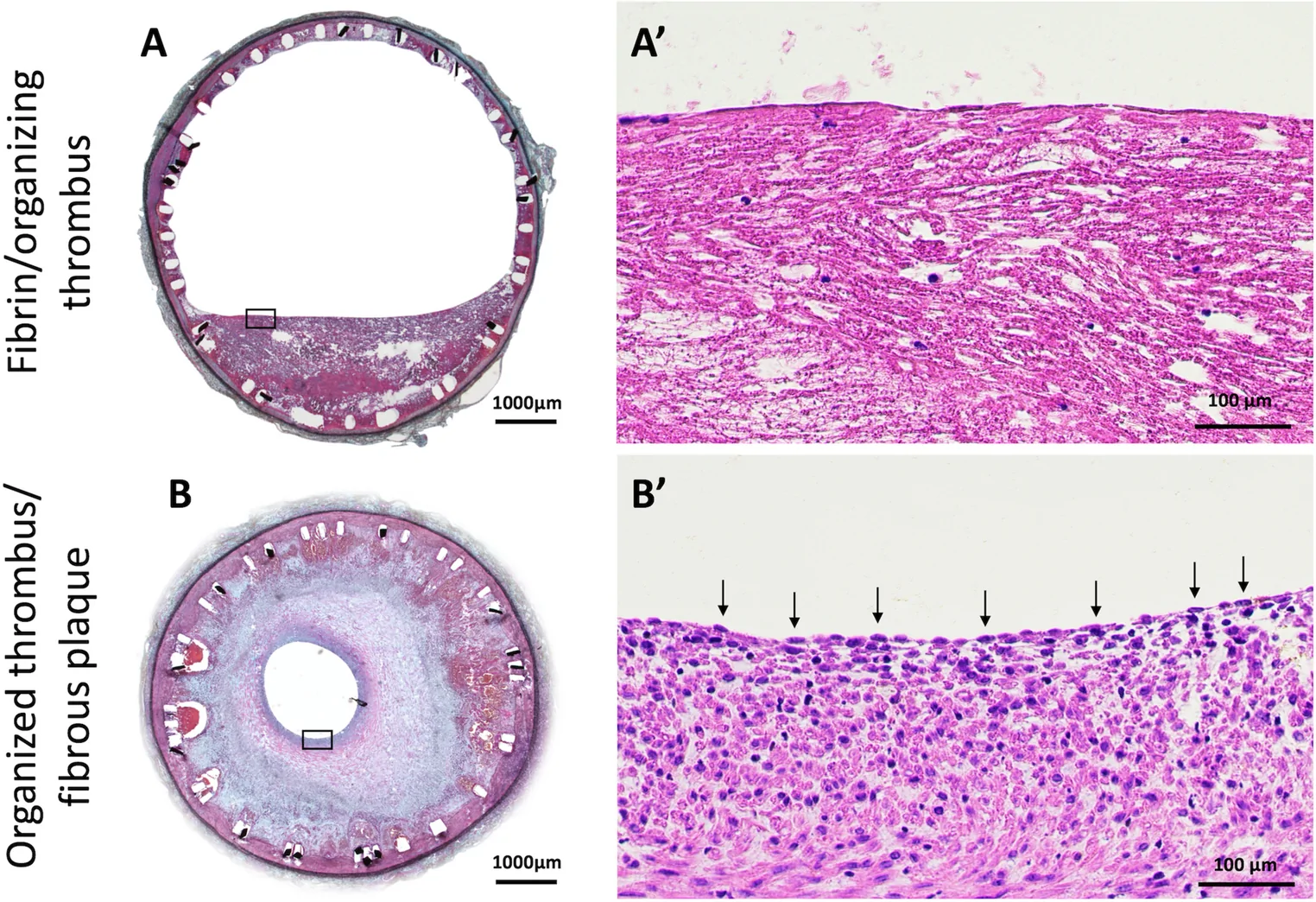
Categories of neointimal tissue. Representative photomicrographs of fibrin/organizing thrombus (**A**) and organized thrombus/fibrous plaque (**B**) stained with Movat Pentachrome. Fibrin/organizing thrombus was defined as fibrin deposition surrounding the stent struts and organized thrombus/fibrous plaque was defined as thrombus with extracellular matrix and/or smooth muscle cells, covered by endothelial cells. High-power images with hematoxylin and eosin staining demonstrate representative images of fibrin/organizing thrombus (**A’**) and organized thrombus/fibrous plaque (**B’**) thrombus. Arrows indicate endothelial cells

Stented arteries were embedded in methyl methacrylate resin. The stented segments were cut at 3 mm intervals, and 4–6 μm thick sections were prepared and stained with hematoxylin and eosin and Movat Pentachrome for light microscopic evaluation. Although the stents were 40 mm in length and sectioned at 3 mm intervals, not all sections were eligible for the final analysis. Sections exhibiting substantial processing artifacts or poor tissue preservation were excluded before image co-registration. The histological characteristics of neointimal tissue were categorized by blinded observers into two patterns: (1) fibrin/organizing thrombus, defined as fibrin deposition surrounding the stent struts; and (2) organized thrombus/fibrous plaque, defined as organized thrombus with extracellular matrix and/or smooth muscle cells covered by endothelial cells [6,19–21] (Fig. 1).

### Image and histology co-registration

**Fig. 2:**
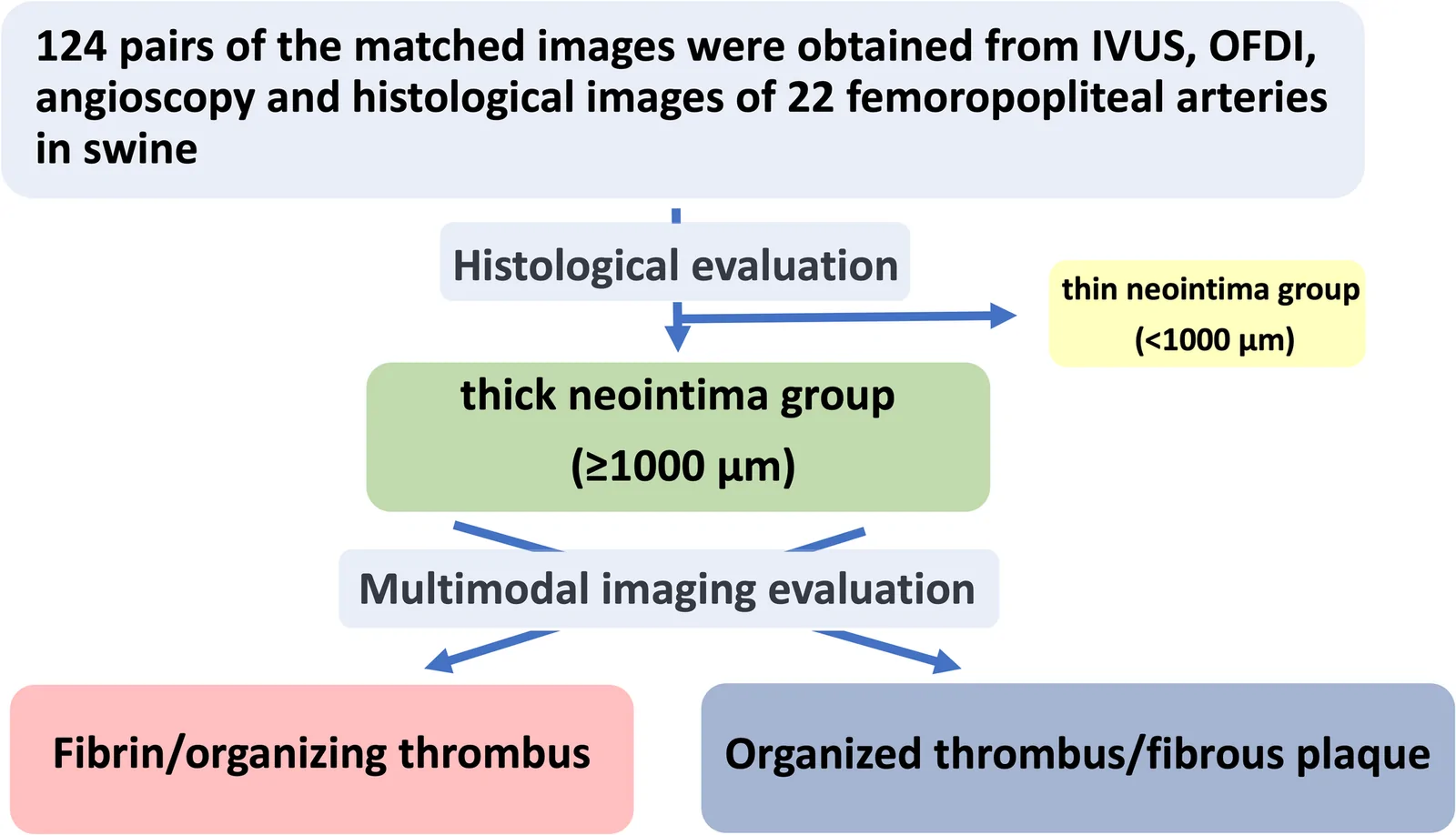
Lesion flowchart for the current study. From the 22 femoropopliteal arteries of swine, 124 pairs of matched images were obtained and evaluated. IVUS: intravascular ultrasound; OFDI: optical frequency domain imaging

To compare imaging with histology, single frames from IVUS and OFDI were matched to corresponding histological images using anatomical landmarks such as vessel branches. Angioscopic images were matched using measured distances from the proximal and distal stent edges. The analysis focused exclusively on segments where the neointimal thickness was ≥ 1000 μm based on histological evaluation, which were defined as the “thick neointima group”, according to the predefined study framework. (Fig. 2) Neointimal thickness was defined as the maximum neointimal thickness measured within each histological section, rather than an average thickness across the segment. The predefined cutoff of 1000 μm was therefore applied to the maximum measured thickness per segment. Only segments that fulfilled all the following predefined criteria were included in the final analysis: (1) adequate histological quality without sectioning artifacts, (2) successful co-registration across IVUS, OFDI, angioscopy, and histology, and (3) histologically confirmed neointimal thickness ≥ 1000 μm. The primary diagnostic accuracy assessment was subsequently performed on the thick neointima group.

### Statistical analysis

Using histological findings as the gold standard, the diagnostic accuracy of each imaging modality was assessed by calculating sensitivity, specificity, positive predictive value (PPV), and negative predictive value (NPV). To account for potential within-animal correlation among multiple histological sections, all diagnostic metrics (sensitivity, specificity, PPV, and NPV) were first summarized on a per-animal basis. Each animal served as an independent observational unit, and the mean values of each metric were calculated across animals. To estimate the variability and 95% confidence intervals (CI) of these metrics while considering within-animal clustering, we performed cluster bootstrap resampling (2,000 iterations) at the animal level. This approach appropriately accounts for the non-independence of observations derived from the same animals and provides a valid CI for diagnostic performance measures. All statistical analyses were performed using JMP software version Student Edition 18.0 (SAS Institute, Cary, North Carolina, USA) and Python software version 1.10.1 (SciPy library; Python Software Foundation, Wilmington, Delaware, USA).

## Results

### Baseline animal and angiographic outcomes

All swine successfully completed the scheduled one-month observation period without significant adverse events. Follow-up angiography confirmed the absence of vessel dissections, ectasia, or aneurysms. Post-fixation radiographs of all stented arteries demonstrated full stent expansion without evidence of fracture. Additionally, all tibial arteries subjected to coil embolization were confirmed to be occluded, with the formation of bridging collateral vessels supplying the affected areas.

### Histological findings and cohort description

A total of 36 femoropopliteal arteries from 18 swine were included in the experimental model. Among these, 124 co-registered images from 22 femoropopliteal arteries in 11 swine met the predefined criteria—namely, adequate histological quality and successful multimodality co-registration—and were included in the analysis. Based on neointimal thickness in histology, these segments were divided into a thick neointima group (*n* = 62) and a thin neointima group (*n* = 62). The primary analysis focused on the thick neointima group, where the causes of in-stent tissue formation were categorized as either fibrin/organizing thrombus or organized thrombus/fibrous plaque. Histological evaluation of this group revealed that 13 of the 62 segments (21%) were predominantly composed of fibrin/organizing thrombus (Table 1).

**Table 1:** Actual interpretation of neointimal characteristics by IVUS, OFDI, angioscopy, and histology in the thick neointima group

Thick neointima group			
		Histology (*n*=62)	
		Fibrin/organizing thrombus	Organized thrombus/fibrous plaque
IVUS	Fibrin/organizing thrombus	2	1
	Organized thrombus/fibrous plaque	11	48
OFDI	Fibrin/organizing thrombus	11	8
	Organized thrombus/fibrous plaque	2	41
IVUS + OFDI	Fibrin/organizing thrombus	10	1
	Organized thrombus/fibrous plaque	3	48
Angioscopy	Fibrin/organizing thrombus	3	0
	Organized thrombus/fibrous plaque	10	49

IVUS = intravascular ultrasound; OFDI = optical frequency domain imaging

### Quantitative characteristics of thick neointimal lesions

Quantitative ex vivo IVUS and OFDI measurements in the thick neointima group are summarized in Table 2. Mean percent area stenosis was 68.3 ± 21.5% by IVUS and 70.6 ± 20.1% by OFDI, indicating that the analyzed lesions represented relatively advanced ISR.

**Table 2:** Quantitative measurements by IVUS and OFDI

	IVUS (*n* = 62)	OFDI (*n* = 62)
Stent area, mm²	23.6 ± 1.5	25.18 ± 2.3
Lumen area, mm²	7.4 ± 4.9	7.5 ± 5.3
Neointimal area, mm²	16.2 ± 5.3	17.7 ± 5.2
Area stenosis, %	68.3 ± 21.5	70.6 ± 20.1

IVUS = intravascular ultrasound; OFDI = optical frequency domain imaging

### Diagnostic accuracy in thick neointima (≥ 1000 μm)

**Fig. 3:**
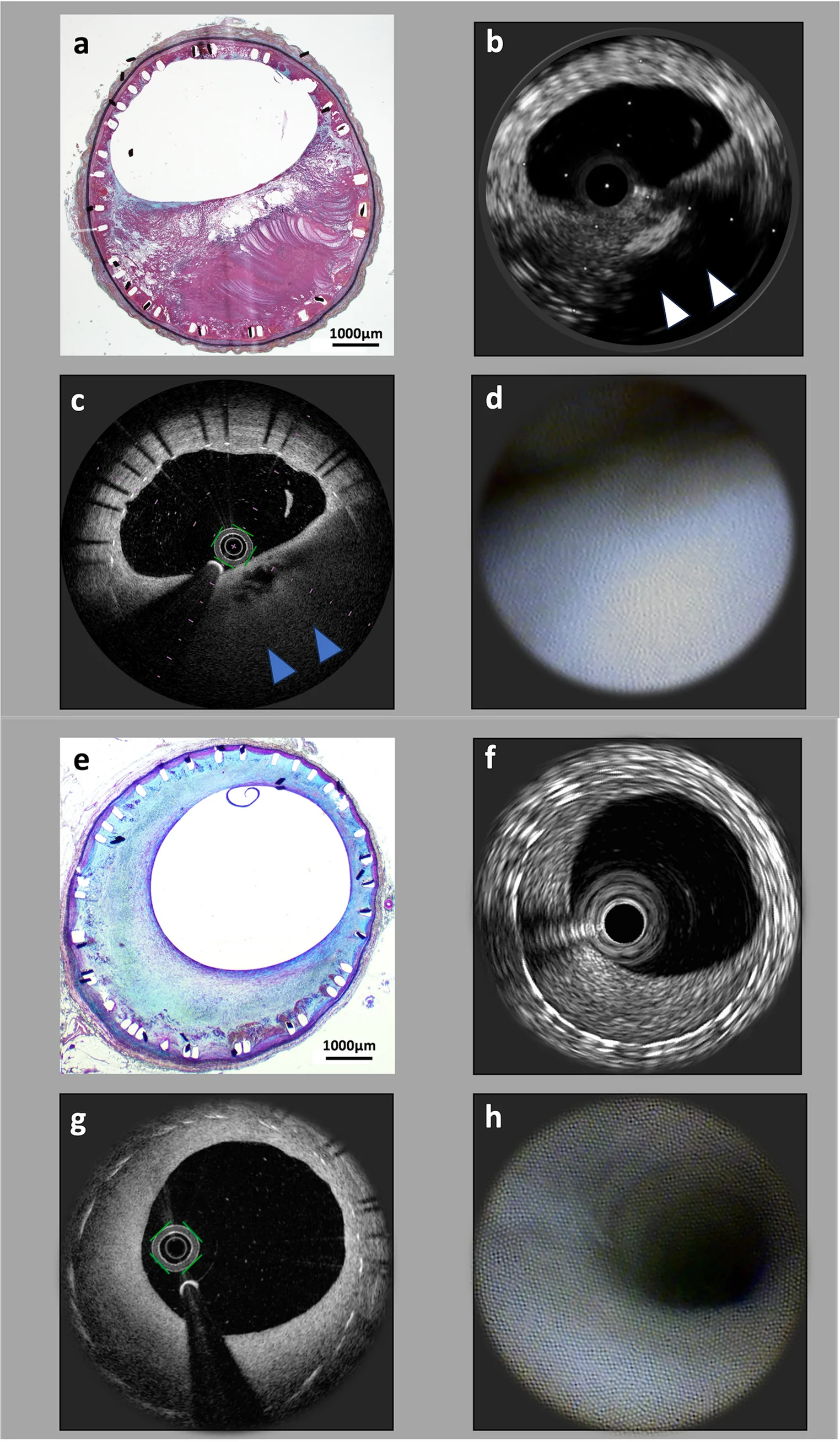
**A**. Representative co-registered images of fibrin/organizing thrombus in the thick neointima group. A histological image was stained with Movat Pentachrome. (**a**) A large amount of fibrin was observed surrounding the stent struts. (**b**) On IVUS observations, fibrin-rich regions appeared as hypoechoic areas (white arrow heads). (**c**) On OFDI observation, fibrin-rich regions exhibited high backscattering with attenuation (blue arrow heads) resulting in invisible struts. (**d**) On angioscopy, the surface of the fibrin/organizing thrombus appeared white. **B.** Representative co-registered images of organized thrombus/fibrous plaque in the thick neointima group. A histological image was stained with Movat Pentachrome. (**e**) The neointima, composed of extracellular matrix, was observed. (**f**) IVUS showed an isoechoic area in the neointima. (**g**) OFDI exhibited homogenous backscattering with low attenuation, allowing the stent struts to remain visible. (**h**) On angioscopy, the surface of the organized thrombus/fibrous plaque appeared white, similar to the fibrin/organizing thrombus IVUS: intravascular ultrasound; OFDI: optical frequency domain imaging

**Fig. 4:**
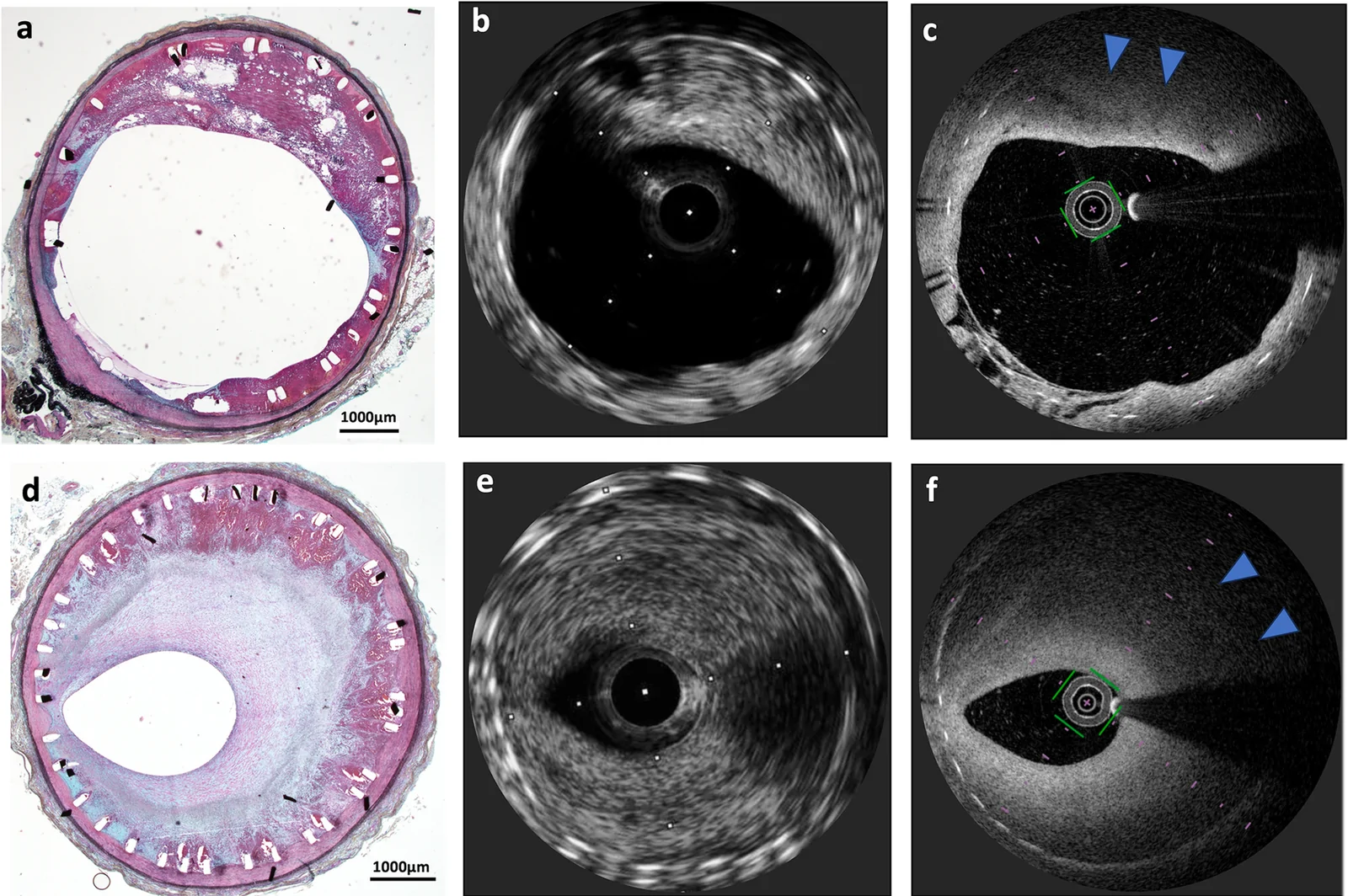
**A**. Representative images demonstrating the underestimation of fibrin/organizing thrombus on IVUS. (**a**) A histological image stained with Movat Pentachrome showed a large amount of fibrin around the stent struts. OFDI demonstrated high backscattering with attenuation in fibrin-rich area (c, blue arrow heads); however, this area appeared isoechoic on IVUS (**b**), leading to an underestimation of fibrin/organizing thrombus. **B**. Representative images demonstrating the overestimation of fibrin/organizing thrombus on OFDI. (**d**) A histological image stained with Movat Pentachrome showed extracellular matrix-rich neointima with some fibrin around the stent struts. IVUS exhibited isoechoic area within neointimal area (**e**); whereas, OFDI showed high backscattering with attenuation (**f**, blue arrow heads), potentially leading to an overestimation of fibrin/organizing thrombus

Using histology as the gold standard, the diagnostic performance of each imaging modality for detecting fibrin/organizing thrombus was assessed in the thick neointima group (Table 1; Fig. 3). IVUS demonstrated a high specificity of 96% (CI: 88.9%–100%) but a very low sensitivity of 13% (CI: 0.0%–33.3%), with a PPV of 75% (CI: 50.0%–100%) and a NPV of 78% (CI: 56.0%–94.8%) (Table 3; Fig. 4A). OFDI showed a relatively high sensitivity of 60% (CI: 20.0%–100%) but a lower specificity of 83% (CI: 61.9%–100%) and a PPV of 50% (CI: 10.0%–90.0%), suggesting a tendency for overestimation (Table 3; Fig. 4B). Its NPV was 94% (CI: 85.2%–100%). Angioscopy yielded a specificity and PPV of 100% (CI: 100%–100%), but its sensitivity was only 24% (CI: 0.0%–63.3%). The paired use of IVUS and OFDI significantly improved diagnostic accuracy, achieving a sensitivity of 50% (CI: 10.0%–90.0%), specificity of 96% (CI: 88.9%–100%), PPV of 83% (CI: 50.0%–100%), and NPV of 93% (CI: 84.4%–98.6%) (Table 3). Notably, no segments demonstrated the discordant pattern of fibrin/organizing thrombus on IVUS and organized thrombus/fibrous plaque on OFDI. The distribution of concordant and discordant IVUS and OFDI findings is summarized in Supplemental Table S1.

**Table 3:** Accuracy of IVUS, OFDI, and angioscopy for fibrin/organizing thrombus in the thick neointima group

Thick neointima group				
	Sensitivity	Specificity	PPV	NPV
IVUS (%)	13 (CI: 0.0–33.3)	96 (CI: 88.9–100)	75 (CI: 50.0–100)	78 (CI: 56.0–94.8)
OFDI (%)	60 (CI: 20.0–100)	83 (CI: 61.9–100)	50 (CI: 10.0–90.0)	94 (CI: 85.2–100)
IVUS + OFDI (%)	50 (CI: 10.0–90.0)	96 (CI: 88.9–100)	83 (CI: 50.0–100)	93 (CI: 84.4–98.6)
Angioscopy (%)	24 (CI: 0.0–63.3)	100 (CI: 100–100)	100 (CI: 100–100)	82 (CI: 58.5–98.0)

CI = confidence interval; IVUS = intravascular ultrasound; NPV = negative predictive value; OFDI = optical frequency domain imaging; PPV = positive predictive value

## Discussion

This study provides the first histological validation of modern intravascular imaging modalities for assessing neointimal tissue characteristics in a swine model of femoropopliteal stent failure. The principal finding is that while IVUS and OFDI each have distinct limitations, their paired use provides a highly accurate assessment of neointimal composition, particularly for differentiating fibrin-rich from organized tissue.

Individually, the modalities showed contrasting profiles. IVUS exhibited high diagnostic specificity for fibrin/organizing thrombus but tended to underestimate its presence, as reflected by its very low sensitivity. This is likely attributable to the limited resolution of IVUS, which may not distinguish subtle textural differences in tissue. Conversely, OFDI demonstrated high sensitivity for detecting fibrin/organizing thrombus but had a lower PPV, suggesting a tendency for overestimation. While OFDI offers superior resolution to IVUS, its limited tissue penetration depth—especially in larger peripheral arteries—may have led to signal attenuation artifacts being misinterpreted as fibrin-rich tissue. These findings suggest that relying on either IVUS or OFDI alone for characterizing in-stent tissue can be misleading.

The paired assessment of IVUS and OFDI, however, overcame these complementary weaknesses and significantly improved diagnostic accuracy. This suggests that hybrid IVUS-OFDI imaging catheters [22, 23], which are currently in development, may offer potential advantages for the assessment of femoropopliteal stent failure. In this study, we also assessed angioscopy, which yielded perfect specificity and PPV for fibrin/organizing thrombus. This suggests that angioscopy is a highly reliable tool for confirming the presence of thrombotic material when it is visualized. However, its application is likely limited by a very low sensitivity, meaning it cannot be used to reliably rule out such lesions. This characteristic is likely because angioscopy can only visualize the endothelial surface of the neointima, not the tissue beneath it.

Our study confirmed that both fibrin/organizing thrombus and organized thrombus/fibrous plaque are key contributors to in-stent tissue buildup. Furthermore, the findings of the present study demonstrated the distinct characteristics of IVUS, OFDI, and angioscopy, highlighting the diagnostic potential of the paired use of IVUS and OFDI for neointimal tissue characterization. The ability to apply a paired-use imaging approach using IVUS and OFDI may also have important clinical implications in femoropopliteal stent failure. Lesions predominantly composed of fibrous neointima may respond favorably to drug-coated balloon treatment, whereas lesions with substantial fibrin or organizing thrombus may be more suitable for excimer laser atherectomy or thrombectomy-based approaches. In addition, identification of thrombotic burden may potentially influence post-procedural antithrombotic management. Therefore, complementary interpretation of IVUS and OFDI findings may facilitate more tailored treatment strategies and improved patient outcomes.

## Study limitations

This study has several limitations. First, it was conducted using the femoropopliteal arteries of healthy swine, which lack human-like biomechanics and atherosclerotic changes, and the histological characteristics of in-stent tissue may not fully replicate those observed in human atherosclerotic arteries. Because this study used a healthy swine model with early follow-up after stent implantation, lipid-rich neointima and calcified components were not observed histologically. Therefore, neointimal tissue characterization was focused on differentiating fibrin/organizing thrombus from organized thrombus/fibrous plaque, which represented the predominant tissue patterns in this model. Second, the ex vivo nature of the experiments precludes the evaluation of imaging performance under live hemodynamic conditions. Although this approach precludes evaluation of hemodynamic influences and may not fully replicate in vivo conditions, the ex vivo design was essential to achieve precise co-registration between imaging modalities and histology, which is virtually unattainable in a clinical setting. In addition, adequate blood clearance—required for both OFDI and angioscopy —is technically difficult to achieve in the high-flow femoropopliteal model in vivo and may introduce variability in image interpretation. Nevertheless, imaging was performed using formalin-fixed specimens, and tissue shrinkage and structural alterations compared with fresh specimens and in vivo conditions may have affected tissue characteristics and the interpretation of intravascular imaging findings. The ex vivo approach enabled standardized visualization across modalities and reduced procedural artifacts, including potential disruption of fragile fibrin deposition during catheter manipulation, thereby allowing a more controlled assessment of stent strut coverage. Third, there was a limited number of evaluated samples (22 femoropopliteal arteries) because of both cost and ethical considerations, and multiple histological sections were analyzed per animal. However, as several previous animal studies were performed with a similar number of samples [11, 24], and the present analysis accounted for within-animal correlation by using cluster bootstrap resampling at the animal level. Therefore, we assumed that the number of arteries in this study was sufficient to evaluate the accuracy of intravascular imaging. Finally, the present study focused primarily on tissue characterization in segments with thick neointima rather than on lesion severity. In addition, the 1000 μm threshold used to define thick neointima was a study-specific classification criterion within the present experimental framework and should not be interpreted as a universally applicable cutoff. Further studies evaluating the relationship between neointimal maturation and stenosis severity may provide additional mechanistic insights.

## Conclusions

This study demonstrated the distinct characteristics of IVUS, OFDI, and angioscopy in assessing neointimal composition and highlighted the complementary value of IVUS and OFDI. Paired use of IVUS and OFDI may improve diagnostic confidence for thick neointima after stent implantation.

## Supplementary Information

Below is the link to the electronic supplementary material.

Supplementary Material 1
