# Supplementary material for: Accuracy of multimodal intravascular imaging in evaluating neointimal tissue in a swine femoropopliteal artery stent model
Source: Cardiovasc Interv Ther. 2026 Jul 29;41(4):1077–86. doi: 10.1007/s12928-026-01321-y (PMC13582157; doi:10.1007/s12928-026-01321-y)
Supplement: Supplementary file 1 [file 12928_2026_1321_MOESM1_ESM.docx]

**Supplemental Table S1.** Cross-tabulation of IVUS and OFDI interpretations in the thick neointima group

| Thick neointima group (n=62) | | | |
| --- | --- | --- | --- |
|  | | OFDI | |
|  |  | Fibrin / organizing thrombus | Organized thrombus / fibrous plaque |
| IVUS | Fibrin / organizing thrombus | 3 | 0 |
|  | Organized thrombus / fibrous plaque | 16 | 43 |

IVUS = intravascular ultrasound; OFDI = optical frequency domain imaging
